# Quality of life and costs of spasticity treatment in German stroke patients

**DOI:** 10.1186/s13561-016-0107-5

**Published:** 2016-07-08

**Authors:** Reinhard Rychlik, Fabian Kreimendahl, Nicole Schnur, Judith Lambert-Baumann, Dirk Dressler

**Affiliations:** 1 Institute of Empirical Health Economics, Am Ziegelfeld 28, D-51399 Burscheid, Germany; 2 Movement Disorders Section, Department of Neurology, Hannover Medical School, Hannover, Germany; 3 Merz Pharmaceuticals, Frankfurt/M, Germany

**Keywords:** Post-stroke spasticity, IncobotulinumtoxinA, Cost-utility, Ashworth score, I10, I120

## Abstract

**Objective:**

To gather data about the medical and non-medical health service in patients suffering from post-stroke spasticity of the upper limb and evaluate treatment effectiveness and tolerability as well as costs over the treatment period of one year.

**Methods:**

Prospective, non-interventional, multicenter, parallel-group study comparing effectivenessand costs of incobotulinumtoxinA (INCO) treatment (*n* = 118) to conventional (CON) antispastic therapy (*n* = 110) for upper limb spasticity after stroke in 47 clinical practices across Germany over a 1-year treatment period. IncobotulinumtoxinA was applied according to the individual treatment algorithms of each participating site and additional antispastic treatments were allowed. Primary efficacy objective was the reduction of the muscle tone measured by Ashworth scale. Responder analyses and logistic regressions were performed. Quality of life, measured by SF-12 questionnaire and functional disability were assessed. Besides calculating treatment costs, a cost-utility analysis was performed.

**Results:**

Responder rates of all muscle groups of the upper extremities were significantly higher in the treatment group (62.9–86.2 % vs. 15.5–26.9 %, *p* < 0.01). Total health service costs were twice as high in the INCO group, however cost-utility ratios were consistently superior compared to the control group. Lowest incremental costs were documented to improve the “physical health” dimension in quality of life.

**Conclusion:**

Higher responder rates, higher increases in quality of life and superior cost-utility ratios in the BoNT/A-treatment group underline guideline recommendations for botulinum toxin A treatment in focal or segmental spasticity. Results may partially be influenced by different patient demographics or disease severity at study entry.

## Background

Spasticity is a disorder of the sensorimotor system characterized by a velocity-dependent increase in muscle tone that interferes with limb positioning, grasping, walking, self-care and other activities of daily living [1, 2]. It is a common complication for stroke survivors and is often more severe in upper than lower limb muscles [2]. Prevalence estimates range from 4 % to 42.6 % with incidences of disabling spasticity from 2 % to 13 % [3]. In Germany, the incidence of stroke is about 150–200 in 100.000. 24 % of patients already show an increased muscle tone in the affected extremities during the first week after stroke [4]. Onset is highly variable and can occur early within the first few weeks or more than a year after stroke. 19–38 % of patients develop spasticity during the first year after stroke. During the course of the disease, contractures may evolve which lead to permanent painful and deformed posture. Starting treatment early might be effective in reducing contracture development and thus functional impairments [5]. In German as well international guidelines and evidence based reviews [6–10], BoNT/A is recommended as a treatment of choice for focal, multifocal and regional spasticity and received level A recommendation for the treatment of post-stroke spasticity [11]. However, botulinum toxin treatment has so far not been implemented in routine health care practice in Germany [12].

This prospective, multicenter, non-interventional parallel-group study was performed to gather routine clinical practice data on post-stroke spasticity patients and their treatments in Germany. Drug treatment as well as non-pharmacological therapies were documented to analyze efficacy, impact on quality of life and costs over a one year treatment period. Conventional therapy was compared to incobotulinumtoxinA (Xeomin®; Merz Pharmaceuticals GmbH, Frankfurt/M, Germany), a BoNT/A preparation free of complexing proteins [13]. Apart from the symptomatic treatment of spasmodic torticollis and blepharospasm, incobotulinumtoxinA is approved in Germany for the treatment of post-stroke spasticity of the upper limb presenting with flexed wrist and clenched fist in adults [14]. IncobotulinumtoxinA is available in many countries worldwide and is approved in US for upper limb spasticity treatment.

## Methods

The prospective, non-interventional, parallel-group study was approved by the ethics committee of Hannover Medical School (Germany) and conducted by the Institute of Empirical Health Economics (Burscheid, Germany) between April 2012 and May 2014 in accordance with the Medicines Act of the Federal Republic of Germany. All participating patients gave written informed consent.

The study compared two patient groups: One arm included patients receiving conventional measures such as oral antispastic medications, physiotherapy and others, patients in the other arm received incobotulinumtoxinA injections plus conventional therapy if required.

Patients in the INCO group were additionally separated in patients who had received any botulinum toxin treatment before study start (‘BoNT/A-pretreated’) and those who were BoNT/A naïve, since pretreatment may have an effect on the efficacy of the study treatment. Due to the non-interventional study design treatment decisions are solely at the discretion of the participating physicians and the decision how to treat a particular patient (in this study with conventional therapy [CON] or with incobotulinumtoxinA [INCO]) must be made before inclusion of the patient in the study. At study start participating sites had chosen their respective treatment group for all their patients.

Only adult patients were eligible for participation if they suffered from post-stroke upper limb spasticity, were able to read, to understand and complete information material as well as a patient questionnaire. Patients were excluded from the incobotulinumtoxinA arm in case of contraindications to BoNT/A preparations, such as infection at the intended injection site or presence of neuromuscular disorders (e.g. myasthenia gravis, Lambert-Eaton syndrome). In case of a prior BoNT/A treatment, the last injection should date back at least 12 weeks before study start.

The observation period covered one year. Treatment in either group was started at Visit 1 (baseline visit) and continued at visits every 12 weeks (visit 2, 3, 4) until the end of observation (visit 5, visit 5b). Since peak effect of botulinum toxin therapy occurs about four to six weeks after the injection additional efficacy assessments in the INCO group took place four weeks after the first, third and fifth visit (visit 1b, visit 3b and visit 5b). For the control group, it was expected that systemic oral antispastic medication will result in a steady treatment effect.

All antispastic medications including incobotulinumtoxinA were prescribed according to the respective summary of product characteristics and physicians discretion.

### Assessments

The primary goal of antispastic therapy is the reduction of increased muscle tone. The 5-point Ashworth scale presents an established and approved tool in the neurological practice [15, 16]. It measures the increase of muscle tone by testing muscle resistance against passive movements performed by the evaluating physician. A reduction by 1 point is considered clinically significant [17, 18].

The primary efficacy parameter was a responder analysis at study end. Responders were defined as patients with a 1-point or higher improvement (reduction) from baseline in the Ashworth score from baseline to the end of the study (visit 5 for the CON group, around 12 months of treatment; visit 5b for the INCO group, around 13 months of treatment). Treatment groups were compared using Fisher’s Exact Test. Only patients with baseline values in Ashworth scale ≥ 1were considered in the analysis. Responder analyses were performed for each of the nine spasticity patterns of upper limb spasticity. Other assessment parameters were overall efficacy and tolerability, both rated by physicians and patients on a 5-point scale. Functional disability was rated by the physician and patient on the Disability Assessment Scale (DAS) consisting of the four domains hygiene, dressing, limb position and pain on a 4-point scale ranging from ‘0 = no disability’ to ‘3 = severe disability’. *P*-values for changes in functional disability over the course of the study were calculated with the Wilcoxon-Mann–Whitney test.

Therapy costs were considered from a societal perspective: Direct costs, indirect costs as well as intangible effects in terms of health related quality of life were assessed quarterly. Costs related to the treatment of post-stroke spasticity were calculated for the 1-year treatment period. Direct costs included: Ambulatory medical treatment (study centre visits, office based physicians visits), diagnostic procedures, drugs, non-pharmacological therapies, hospitalizations and other medical interventions (e.g. rehabilitation measures), medical devices/therapeutic aids and nursing home care. Cost data sources are listed in Table 1. Therapy costs are paid by the statutory or private health insurances of the patient. Indirect costs included loss of productivity caused by post-stroke spasticity for patients capable of gainful employment. This includes days absent from work and continuation of remuneration and were valued monetarily based on the average income in Germany.

**Table 1:** Cost data sources

Item	Cost sources
Drugs	German Rote Liste 2013, web-based research
Ambulatory medical treatment	German value measurement (EBM 2000+) and fee regulations for doctors (GOÄ)
Non-pharmacological therapies	According to agreements between German health insurance funds and professional organizations
Medical devices/aids	Web-based research
Hospitalisation and Rehabilitation	German Diagnosis-Related Groups (G-DRG), web- and phone-based research
Nursing home care	According to German long term care insurance
Reduction in earning capacity	Average payments according to German retirement insurance

Intangible effects refer to mental and physical burdens patients suffer from and can hence not be measured monetarily. They include pain, depression, social pressure or limited mobility and are expressed by a rating of quality of life. To evaluate intangible effects quality of life was assessed with the “SF-12 v2 Health Survey”, a shortened version of the SF-36 [19]. The SF-12 includes 12 items, which are subsumed in two dimensions ‘mental health’ and ‘physical health’. SF-12 scores in this study are calculated using a fixed algorithm and are based on the analysis manual of the German norm from 1994. Possible scores in both domains range from 0 to 100, high scores indicating a high quality of life. Comparisons between baseline and end observation employed the paired *t*-test.

Utility values in this study were responder rates in Ashworth scale in the respective spasticity pattern and changes in SF-12 scores in mental and physical health after 1-year of antispastic treatment. Cost-utility ratios were calculated by dividing yearly total costs per patient by responder rates as well as the change in SF-12 scores (utility values). The Incremental Cost-utility ratios (ICER) was subsequently assessed according to the formula: ICER = (Total costs INCO – Total costs CON)/(Utility value INCO – Utility value CON). The ICER permits the comparison of relative cost-effectiveness between the treatment groups.

## Results

A total of 218 patients were analysed. 108 patients received incobotulinumtoxinA treatment, 110 patients were treated with conventional therapeutic measures. 62 % in the INCO group had received Botulinum neurotoxin prior to study entry.

The majority of patients were male (58.7 %). The average age at baseline was 64.8 years (SD = 13.1). Patients in the INCO group were about 6 years younger than in the control group. First diagnosis of stroke and post-stroke spasticity had occurred about 2 years earlier in the INCO group (see Table 2). The majority of patients (82.6 %) suffered from concomitant diseases, most commonly from high blood pressure (71.1 %), epilepsy (27.2 %) and depression (26.1 %).

**Table 2:** Patient demography and other baseline characteristics

	INCO pretreated *N =* 67	INCO naïve *N =* 41	INCO total *N =* 108	CON *N =* 110	Total *N =* 218
Gender (m)	36 (53.7 %)	22 (53.7 %)	58 (53.7 %)	70 (63.6 %)	128 (58.7 %)
Age (years)	62.3 (10.7)	60.7 (16.0)	61.7 (12.9)	67.8 (12.7)	64.8 (13.1)
Body mass index (kg/m^2^)	26.7 (4.0)	26.8 (4.4)	26.7 (4.1)	27.7 (4.8)	27.2 (4.5)
Time since apoplex (years)	8.0 (5.6)	6.8 (6.1)	7.5 (5.8)	5.3 (5.1)	6.5 (5.6)
Time since spasticity (years)	6.9 (6.3)	6.0 (6.5)	6.6 (6.3)	4.9 (5.4)	5.7 (5.9)
Concomitant diseases (yes)	55 (82.1 %)	29 (70.7 %)	84 (77.8 %)	96 (87.3 %)	180 (8.6 %)
Employed (yes)	1 (1.5 %)	3 (7.3 %)	4 (3.7 %)	9 (8.2 %)	13 (6.0 %)
Retired (yes)	58 (96.7 %)	39 (97.5 %)	87 (94.6 %)	86 (86.9 %)	173 (90.6 %)
Early retirement due to spasticity (yes)	40 (63.5 %)	20 (55.6 %)	60 (60.6 %)	19 (20.4 %)	79 (41.1 %)
Reduction in earning capacity due to spasticity (yes)	22 (32.9 %)	11 (26.8 %)	33 (30.6 %)	23 (20.9 %)	56 (25.7 %)
Level of care (none)	12 (17.9 %)	10 (24.4 %)	22 (20.4 %)	37 (35.6 %)	59 (27.8 %)
Level 1	28 (41.8 %)	15 (36.6 %)	43 (39.8 %)	33 (31.7 %)	76 (35.8 %)
Level 2	23 (34.3 %)	15 (36.6 %)	38 (35.2 %)	29 (27.9 %)	67 (31.6 %)
Level 3	4 (6.0 %)	1 (2.4 %)	5 (4.6 %)	5 (4.8 %)	10 (4.7 %)

All values are means (± standard deviation) or number of patients (%)

At the time of study start only 6.0 % of the patients were still employed, most patients were retired (90.6 %). 20.4 % (CON group) and 60.6 % (INCO group) of the patients had retired earlier due to spasticity. More patients in the INCO group (79.6) were in need of care than in the CON group (64.4 %).

About every second patient (56.9 %) suffered from spasticity of both the upper and lower limb. 54.2 % in the INCO group and 32.1 % in the CON group suffer from upper limb spasticity only (total: 43.1 %). The most frequent clinical patterns of spasticity of the upper limb were flexed elbow (85.8 % of all patients), flexed wrist (71.6 %), shoulder adduction/internal rotation (71.1 %) and clenched fist and forearm pronation (both 70.6 %).

### Antispasticity treatment

In the incobotulinumtoxinA group, 38 % of the patients were BoNT/A naïve. The remainder had received their last BoNT/A injection a median 15.7 weeks (range 12–171 weeks) before start of study. Mean incobotulinumtoxinA doses at first injection were 215 ± 114 U, at last (5^th^) injection 268.7 ± 155 U. Prior to start of study 38 patients in this group (35.2 %) had received oral antispasticity medication (mainly baclofen [63.2 %] and tolperisone [21.1 %]). Thirty-seven (33.6 %) of the patients in the conventional therapy arm had received oral antispasticity medication prior to start of study (mainly baclofen [62.2 %], tetrazepam [16.2 %], and tolperisone [10.8 %]). During the study physicians documented oral antispastic medication in 19.1–32.4 % of patients in the INCO-group and 60.9–69.2 % in the CON-group depending on the treatment quarter (see Table 3). Most commonly prescribed in the INCO group was baclofen (48.4–70 % of patients with oral medication), in the CON group baclofen (45.6–49.3 %) and tetrazepam (10.5–17.9 %). The proportion of patients receiving any physiotherapy was slightly higher in the CON group (54.5–61.9 % vs. 52.9–55.6 %); the proportion of patients receiving occupational therapy was markedly higher in the incobotulinumtoxinA group (39.8–46.8 % vs. 9.5–13.6 %). The use of additional therapeutic aids such as ortheses etc. was markedly reduced in the last 3 months of incobotulinumtoxinA treatment.

**Table 3:** Overview of antispastic therapies and measures during the study

IncobotulinumtoxinA group: Antispastic medications except BoNT/A, non-pharmacological therapies and aids				
	First quarter (*n* = 108)	Second quarter (*n* = 102)	Third quarter (*n* = 99)	Fourth quarter (*n* = 94)
Oral medication	31 (28.7 %)	23 (32.4 %)	20 (20.2 %)	18 (19.1 %)
Physical therapy	60 (55.6 %)	54 (52.9 %)	54 (54.5 %)	51 (54.3 %)
Occupational therapy	43 (39.8 %)	42 (41.2 %)	41 (41.4 %)	44 (46.8 %)
Speech therapy	10 (9.3 %)	8 (7.8 %)	8 (8.1 %)	9 (8.6 %)
Other therapies	3 (2.8 %)	6 (6.0 %)	4 (4.0 %)	4 (4.3 %)
Therapeutic aids	12 (11.0 %)	5 (5.7 %)	-	1 (1.0 %)
Conventional therapy group: Antispastic medications, non-pharmacological therapies and aids				
	First quarter (*n* = 110)	Second quarter (*n* = 98)	Third quarter (*n* = 91)	Fourth quarter (*n* = 84)
Oral medication	67 (60.9 %)	66 (67.3 %)	63 (69.2 %)	58 (69.0 %)
Physical therapy	68 (61.8 %)	59 (60.2 %)	54 (54.5 %)	52 (61.9 %)
Occupational therapy	15 (13.6 %)	11 (11.2 %)	11 (12.1 %)	8 (9.5 %)
Speech therapy	5 (4.6 %)	5 (5.1 %)	5 (5.5 %)	4 (4.8 %)
Other therapies	3 (2.7 %)	5 (5.1 %)	-	1 (1.2 %)
Therapeutic aids	10 (11.0 %)	8 (8.2 %)	12 (13.2 %)	8 (9.5 %)

### Responder analyses

Results of the primary efficacy parameter, responder analyses in Ashworth score from visit 1 to the end of the study demonstrate clinically highly meaningful changes in the INCO group compared to baseline for all clinical patterns (see Table 4). BoNT/A-naïve benefit more than pretreated patients. The results were highly significant for all group comparisons between incobotulinumtoxinA treatment and conventional antispastic treatment (Fisher´s exact test, *p* < 0.01). The highest responder rate with conventional therapy was 26.9 % (flexed elbow), whereas in the INCO group, the lowest responder rate was 56.4 % (shoulder adduction/internal rotation). Logistic regressions (data not shown) indicate, that no significant influence of factors such as sex, age, BMI, time since diagnosis of stroke and spasticity and Ashworth Scale score on baseline exist. These findings indicate that there is no influence of external factors on therapeutic success.

**Table 4:** Responder analyses at study end after 1-year of treatment

	INCO pretreated	INCO naïve	INCO total	CON	INCO pretr. vs. CON	INCO naïve vs. CON	INCO total vs. CON
Shoulder adduction/internal rotation	56.4	73.9	62.9	15.5	<0.01	<0.01	<0.01
Shoulder abduction	65.5	100	73.0	19.7	<0.01	<0.01	<0.01
Shoulder elevation	66.7	88.9	72.7	20.6	<0.01	<0.01	<0.01
Flexed elbow	78.3	92.9	83.8	26.9	<0.01	<0.01	<0.01
Forearm pronation	81.4	73.7	79.0	22.0	<0.01	<0.01	<0.01
Flexed wrist	82.1	94.7	86.2	26.6	<0.01	<0.01	<0.01
Thumb-in-palm	77.8	81.3	78.8	20.0	<0.01	<0.01	<0.01
Clenched fist	79.1	95.2	84.4	22.2	<0.01	<0.01	<0.01
Intrinsic-plus-position of the hand	73.3	100	78.9	19.5	<0.01	<0.01	<0.01

Responder rates (%); response was definded as ≥ 1-point improvement on the Ashworth Scale for all treated muscle groups at study end; Fisher’s exact test was used for group comparisons

### Efficacy, tolerability and compliance

At study end the overall efficacy of antispastic therapy was rated as ‘very good’ or ‘good’ by physicians for 90.0 % of patients in the INCO group, but only for 30.7 % in the CON group. Similarly 91.8 % of the physicians rated the tolerability of the treatment with incobotulinumtoxinA as ‚very good’, but only 13.3 % of the CON group. For 62.7 % of the patients in this group the physicians rated the tolerability as “good”. Adherence of the patients to the antispastic therapy is rated as ‘very good’ by physicians for 81.8 % patients of the INCO group, whereas the respective value in the control group is 21.3 %.

### Functional disability

In all four domains of the 4-point scale DAS significant improvements from baseline to study end were found in the INCO group (mean changes for hygiene: −0.7 ± 1.1, dressing −0.8 ± 1.1; limb position −1.0 ± 0.9, pain −0.8 ± 0.9; all *p* < 0.01, Wilcoxon-Mann–Whitney test). In the CON group statistically significant changes could only be demonstrated for “hygiene” (−0.2 ± 0.8) and “limb position” (−0.3 ± 0.7) (both *p* < 0.01).

### Quality of life

**Fig. 1:**
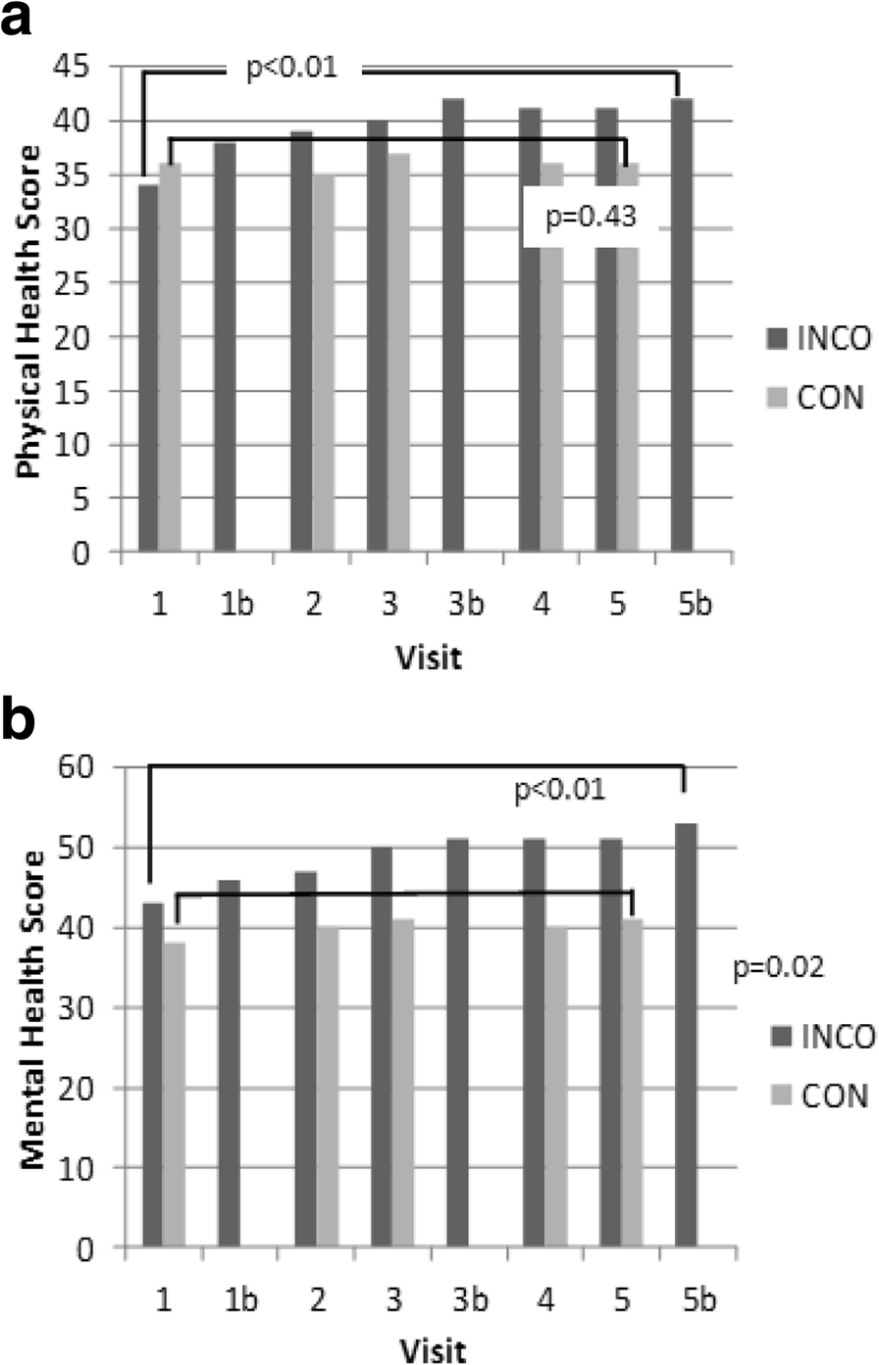
SF-12 – Dimensions ’Physical Health‘ and ‘Mental Health’. **a** Physical Health Score from baseline to study end. **b** Mental Health Score from baseline to study end. Mean values; p-values: change from baseline to study end, one-sample *t*-test for dependent samples

Patients in the CON group (mean baseline score 35.5 ± 9.3) disposed marginally better health state values with respect to ‘physical health’ at baseline than patients in the INCO group (mean baseline score 33.6 ± 7.8). In the dimension ‘mental health’ baseline values were slightly higher in the INCO group (mean baseline score 42.8 ± 14.8) than in the CON group (mean baseline score 37.8 ± 14.4) (see Fig. 1).

After one year of antispastic therapy the INCO group demonstrated clear and significant improvement in mean physical health score by 8.0 ± 8.6 (*p* < 0.01), compared to a change of 0.8 ± 7.9 (*p* = 0.43) in the CON group. ‘Mental health’ score improved significantly in both groups, however stronger in the INCO group: 10.8 ± 16.2 (*p* < 0.01) compared to 5.7 ± 13 (*p* = 0.02). Changes in patients receiving incobotulinumtoxinA increased significantly (*p* < 0.01) independent from their BoNT/A pretreatment status (data not shown).

### Adverse events and adverse drug reactions

A total of 81 Adverse Events (AE) were documented, of which 43 were classified as Serious Adverse Events (SAE) (21 INCO, 22 CON). Testing of the proportion of AEs and SAEs by groups yielded no statistically significant differences between groups (*p* = 0.439 resp. *p* = 0.452). There was no causal relation of AEs to the study medication. Adverse Drug Reactions (ADR) occurred only in one patient (male, 72 years) in the INCO group that reported a mild loss of strength in the left arm.

### Costs

The highest costs in the INCO group were caused by drugs (3,386 €) followed by costs of nursing care (3,089 €). Each of these cost centers account for about one third of total therapy costs (see Table 5). According to the documented treatment rates costs for non-pharmacological therapies in this group were higher than in the CON group. The average amount of drug costs in the CON group is very low and amounts to 193 €. About 50 % of total direct costs in the CON group is composed by costs in nursing care. Overall total costs, including reduction in earning capacity, are about twice as high in the INCO group as compared to the CON group (10,268 € vs. 4,794 €).

**Table 5:** Overview of total costs by cost centers (in €)

	INCO *n* = 93	CON *n* = 83
Ambulatory medical treatment	175	217
Drugs	3,386	193
Hospitalizations (including rehabilitation measures)	40	138
Non-pharmacological therapies	1,408	998
Medical devices/aids	79	12
Nursing home care	3,089	2,203
Total direct costs	8,188	3,806
Reduction in earning capacity	2,081	988
Total costs	10,268	4,794

### Cost-utility analysis

Despite costs being twice as high for patients treated with incobotulinumtoxinA the INCO group shows more favorable cost-utility ratios for every clinical pattern evaluated in the responder analysis of change of muscle tone (compare Table 6). The highest cost-utility ratio in the INCO group (shoulder adduction/internal rotation, 16,325 €) remains below the lowest cost-utility ratio in the CON group (flexed elbow, 17,821 €). This result is based on the responder rates, which are on average three times higher in the INCO group, than in the CON group reaching only placebo level. The best cost-utility ratios for patients treated with incobotulinumtoxinA can be found for the spastic patterns clenched fist, intrinsic-plus position of the hand and the thumb-in-palm position. Only with regards to the SF-12 dimension ‘mental health’ the CON group showed a more favorable cost-utility ratio. Lowest incremental costs at all were calculated for the gain in “physical health” in SF-12 in INCO group.

**Table 6:** Overview of cost-utility ratios and ICER

Utility parameter	INCO		CON		ICER
Responder rate in Ashworth Score per clinical pattern	Responder rate	Cost-utility ratio	Responder rate	Cost-utility ratio	
Shoulder adduction/internal rotation	62.9 %	16,325 €	15.5 %	30,929 €	11,549 €
Shoulder abduction	73.0 %	14,066 €	19.7 %	24,335 €	10,271 €
Shoulder elevation	72.7 %	14,124 €	20.6 %	23,272 €	10,507 €
Flexed elbow	83.8 %	12,253 €	26.9 %	17,821 €	9,621 €
Pronated forearm	79.0 %	12,998 €	22.0 %	21,791 €	9,604 €
Flexed wrist	86.2 %	11,912 €	26.6 %	18,022 €	9,185 €
Thumb-in-palm	78.8 %	13,031 €	20.0 %	23,970 €	9,310 €
Clenched fist	84.4 %	12,166 €	22.2 %	21,595 €	8,801 €
Intrinsic-Plus-position (hand)	78.9 %	13,014 €	19.5 %	24,585 €	9,216 €
Improvement in SF-12 dimension	Improvement	Cost-utility ratio	Improvement	Cost-utility ratio	ICER
Physical Health	7.96	1,290 €	0.83	5,776 €	768 €
Mental Health	10.75	955 €	5.71	840 €	1086 €

Incremental Cost-Effectiveness Ratio (ICER) = (Total costs INCO – Total costs CON)/(Utility value INCO – Utility value CON)

## Discussion

To our knowledge this study is the first evaluation of cost-effectiveness of incobotulinumtoxinA in comparison to conventional antispastic treatment of upper limb spasticity in Germany.

In summary incobotulinumtoxinA proved more effective than conventional therapy in the treatment of upper limb spasticity over a 1-year treatment period in routine clinical practice. Compared to conventional therapy muscle tone decreased significantly (responder rates between 62.9–86.2 %, *p* < 0.01), connected to significant improvements in functional disability (all DAS domains, *p* < 0.01) and quality of life (both SF-12 dimensions mental and physical health, *p* < 0.01) after 1-one year of treatment. Conventional therapy with responder rates between 15.5 % and 26.9 % from baseline to study end, only partially resulted in functional improvements and improved quality of life; changes in DAS domains “hygiene” and “limb position” (*p* < 0.01) and mental health dimension of SF-12 (*p* = 0.02) were significant from baseline in this group.

The non-interventional design was chosen to describe the clinical routine practice in spasticity treatment in different sites and with different treatment options in Germany. Group comparisons of non-interventional parallel-group studies might be biased to the non-randomized patient selection, however the big advantage of this trial design is the description of current treatment situation, effectiveness of treatments and consecutive health care costs.

Spasticity should be treated in a multidisciplinary team approach including physical and occupational therapies as well as antispastic medications. Physiotherapy is recommended as basic treatment for all types of spasticity, botulinum toxin as the treatment of choice for focal, multifocal and segmental spasticity. Particularly in stroke patients coexisting muscle weakness may be aggravated by systemic anticholinergic medications and is therefore only recommended as adjuvant therapy, in particular for severe general spasticity in immobile patients [7]. However, these guideline recommendations have so far not been implemented in routine health care practice in Germany [12]. An analysis of German statutory health insurance data revealed a decrease of patients with post-stroke spasticity receiving physiotherapy after transition into ambulatory care (48 % vs. 89 % for inpatient care). Oral muscle relaxants were used in 13 % of the patients in this database; none received intrathecal baclofen or even botulinum toxin [12].

The results of the primary efficacy outcome, the responder analysis of muscle tone reduction, clearly demonstrate the superiority of the treatment approach in the INCO group with regular incobotulinumtoxinA injections. Responder rates were significantly higher in all measured muscle groups than with conventional therapy alone. The results are comparable to a placebo-controlled trial [20] and its corresponding long-term open-label incobotulinumtoxinA study [21]. In both trials stable antispastic medication and physical and occupational therapy regimens were permitted. The results are also in line with long-term investigations with other BoNT/A preparations in the treatment of spasticity of various etiologies including stroke [22–24].

Higher levels of care at study baseline and rates of early retirement indicate that patients in the INCO group were more severely affected than the CON group. Though patients were slightly younger in the INCO group their diagnosis of stroke and spasticity had occurred earlier than in the CON group. Nevertheless a logistic-regression analysis does not show a meaningful influence of age, disease duration, different Ashworth scores at baseline or any other factors including gender, body size and weight, BMI and treatment group on treatment response.

The burden of spasticity in surviving stroke patients is substantial. Spasticity leads to functional disability in daily living and reduces quality of life. In a German prospective cohort study [3] with acute stroke patients, those who developed spasticity 6 months later showed a lower mean score in EQ-5D (*n* = 75, mean 53.6) compared to patients without developing spasticity (*n* = 80, mean, 62.7; *p* < 0.001). In another study with 66 stroke survivors the impact of spasticity on QoL was determined 18 months after stroke [25]. Patients with spasticity (*n* = 13) had significantly lower scores on the physical function domain of SF-36 (*p* < 0.01) compared to those without spasticity (*n* = 28). In a large retrospective analysis of 328 stroke survivors those patients with spasticity had significant lower SF-12 scores (physical component) and EQ-5D scores (*p* < 0.05) compared to those not reporting spasticity [2, 26]. Increased functional impairment measured with DAS in patients with upper limb post-stroke spasticity was also found to be associated with diminished QoL measured with EQ-5D score [27].

At present only few data exist describing the costs specific to post-stroke spasticity treatment. In the first year after stroke patients with spasticity cause four times higher direct costs compared to patients without spasticity [28]. The primary drivers in costs were hospital care and help at home/residential care. Increased costs were strongly associated with worsening functional ability (r_s_ = 0.624, *p* < 0.001) and with increasing muscle tone (r_s_ = 0.524, *p* < 0.001).

Total costs in the INCO group are about twice as high as in the CON group. Particularly drug costs accounted for about one third of total costs in the INCO group, but for less than 5 % in the control group. Other major contributors to higher costs in the INCO group were nursing home care, accounting for another third of total costs, and the reduction in earning capacity accounting for about 20 %. At study baseline 60.6 % of patients in the INCO group, but only 20.4 % in the CON group were prematurely retired due to spasticity. Additionally 79.6 % of INCO patients compared to 64.4 % of CON patients received payments from nursing care insurance due to documented levels of care needed resulting in higher nursing home care costs.

The total costs in relation to efficacy results of the two treatment groups, the cost-utility ratios, demonstrate consistently better results for the INCO group. Best results were achieved with regards to the “physical health” dimension of quality of life score SF-12, since patients with only conventional therapy failed to improve their “physical health”. The cost-utility ratio for INCO was 4.5-times smaller than for the CON group. The only cost-utility ratio with more favorable results for conventional treatment compared to INCO (840 € vs. 955 €) was to the one for the “mental health” dimension of the SF-12.

Only few studies have so far been conducted to evaluate cost-effectiveness of antispasticity treatment and in particular cost-effectiveness of botulium toxin treatment.

The BoTULS trial investigated cost-effectiveness of abobotulinumtoxinA (Dysport®) plus a specified 4-week upper limb therapy program (*n* = 170) compared to the therapy program alone (*n* = 163) in a randomized controlled setting in UK over a 1-year period [28, 29]. Arm function was measured by the Action Research Arm Test (ARAT) assessing “active” arm function. There was no significant difference between the groups for the primary outcome of improved arm function after one month of treatment (19.5 % control group vs. 25.1 % intervention group, *p* = 0.232). Health related quality of life was assessed using EuroQoL (EQ-5D). Differences between the groups favoring BoNT/A treatment were only found in EQ-5D domain “pain” 3 months after study start, and “anxiety/depression” 12 months after study start. Cost-effectiveness analysis only covered three months from randomization. The overall mean costs per participant were higher in the botulinum toxin group, although the difference was not significant. Biggest contributor to total costs for both groups was costs for other health care and social services contacts. The base case incremental cost-effectiveness ratio for botulinum toxin A plus therapy was 93,500£ per QALY gained.

The international onabotulinumtoxinA (Botox®) economic spasticity trial evaluated patient outcomes and costs of onabotulinumtoxinA plus standard care versus standard care alone in a randomized double-blind placebo-controlled trial design with an open-label extension period in focal upper and lower limb spasticity after stroke [30, 31]. The primary endpoint was the number of patients who achieved their investigator-rated principal active functional goal measured by Goal Attainment Scaling 10 weeks after their second injection at week 24. Similarly to the BoTULS trial the proportion of patients achieving their principal active functional goal with onabotulinumtoxinA plus standard of care was not statistically different from placebo plus standard of care. However, secondary passive functional goal achievement differed significantly at week 24: 60.6 % of patients in the treatment group and 38.6 % in the control group (*p* = 0.016), achieved the passive functional goal. Results of changes in EQ-5D, treatment costs or cost-effectiveness analysis have not been published so far.

In our study incobotulinumtoxinA treatment demonstrated superior results in muscle tone reduction compared to conventional therapy and significantly improved functional impairment as well as quality of life. One of the main cost drivers in the INCO group were drug costs. However cost-utility analysis clearly favored incobotulinumtoxinA treatment in comparison to conventional therapy alone and is recommended with level A in national and international guidelines.

## Conclusions

Due to different trial designs, national varying health care systems and different approaches for calculating cost-effectiveness the comparison of health economic studies is hardly possible. In our study, which collected data from routine clinical practice incobotulinumtoxinA treatment demonstrated superior results in muscle tone reduction compared to conventional therapy and significantly improved functional impairment as well as quality of life. One of the main cost drivers in the INCO group were drug costs. However cost-utility analysis clearly favored incobotulinumtoxinA treatment in comparison to conventional therapy alone. In our view the results underline the level A recommendation of national and international guidelines for treatment of post-stroke spasticity with botulinum toxin. The reasons are unclear, but the treatment rates in this study indicate, that spasticity treatment according to guidelines seems not comprehensively implemented in Germany.

## Abbreviations

ADR, adverse drug reaction; AE, adverse event; ARAT, action research arm test; BoNT/A, botulinum toxin type A; CON, conventional antispastic therapy; DAS, disability assessment scale; EBM, Einheitlicher Bewertungsmassstab (doctor’s fee scale); G-DRG, German Diagnosis-related Groups; GOÄ, Gebührenordnung für Ärzte (medical fee index); ICER, incremental cost-effectiveness ratio; INCO, incobotulinumtoxinA; QoL, quality of life; SAE, serious adverse event
