# Erratum to: ‘Quality of life and costs of spasticity treatment in German stroke patients’

**DOI:** 10.1186/s13561-016-0114-6

**Published:** 2016-08-31

**Authors:** Reinhard Rychlik, Fabian Kreimendahl, Nicole Schnur, Judith Lambert-Baumann, Dirk Dressler

**Affiliations:** 1 Institute of Empirical Health Economics, Am Ziegelfeld 28, D-51399 Burscheid, Germany; 2 Movement Disorders Section, Department of Neurology, Hannover Medical School, Hannover, Germany; 3 Merz Pharmaceuticals, Frankfurt/M, Germany

Unfortunately, the original version of this article [1] contained errors. There were formatting errors in the main text and in Tables 1, 2, 3, 4, 5 and 6. These tables have been included correctly below. There will also be an update to correct the errors in the main text.

**Table 1:** Cost data sources

Item	Cost sources
Drugs	German Rote Liste 2013, web-based research
Ambulatory medical treatment	German value measurement (EBM 2000+) and fee regulations for doctors (GOÄ)
Non-pharmacological therapies	According to agreements between German health insurance funds and professional organizations
Medical devices / aids	Web-based research
Hospitalisation and Rehabilitation	German Diagnosis-Related Groups (G-DRG), web- and phone-based research
Nursing home care	According to German long term care insurance
Reduction in earning capacity	Average payments according to German retirement insurance

**Table 2:** Patient demography and other baseline characteristics

	INCO pretreated *N* = 67	INCO naïve *N* = 41	INCO total *N* = 108	CON *N* = 110	Total *N* = 218
Gender (m)	36 (53.7 %)	22 (53.7 %)	58 (53.7 %)	70 (63.6 %)	128 (58.7 %)
Age (years)	62.3 (10.7)	60.7 (16.0)	61.7 (12.9)	67.8 (12.7)	64.8 (13.1)
Body mass index (kg/m^2^)	26.7 (4.0)	26.8 (4.4)	26.7 (4.1)	27.7 (4.8)	27.2 (4.5)
Time since apoplex (years)	8.0 (5.6)	6.8 (6.1)	7.5 (5.8)	5.3 (5.1)	6.5 (5.6)
Time since spasticity (years)	6.9 (6.3)	6.0 (6.5)	6.6 (6.3)	4.9 (5.4)	5.7 (5.9)
Concomitant diseases (yes)	55 (82.1 %)	29 (70.7 %)	84 (77.8 %)	96 (87.3 %)	180 (8.6 %)
Employed (yes)	1 (1.5 %)	3 (7.3 %)	4 (3.7 %)	9 (8.2 %)	13 (6.0 %)
Retired (yes)	58 (96.7 %)	39 (97.5 %)	87 (94.6 %)	86 (86.9 %)	173 (90.6 %)
Early retirement due to spasticity (yes)	40 (63.5 %)	20 (55.6 %)	60 (60.6 %)	19 (20.4 %)	79 (41.1 %)
Reduction in earning capacity due to spasticity (yes)	22 (32.9 %)	11 (26.8 %)	33 (30.6 %)	23 (20.9 %)	56 (25.7 %)
Level of care (none)	12 (17.9 %)	10 (24.4 %)	22 (20.4 %)	37 (35.6 %)	59 (27.8 %)
Level 1	28 (41.8 %)	15 (36.6 %)	43 (39.8 %)	33 (31.7 %)	76 (35.8 %)
Level 2	23 (34.3 %)	15 (36.6 %)	38 (35.2 %)	29 (27.9 %)	67 (31.6 %)
Level 3	4 (6.0 %)	1 (2.4 %)	5 (4.6 %)	5 (4.8 %)	10 (4.7 %)

All values are means (± standard deviation) or number of patients (%)

**Table 3:** Overview of antispastic therapies and measures during the study

IncobotulinumtoxinA group: Antispastic medications except BoNT/A, non-pharmacological therapies and aids				
	First quarter (n = 108)	Second quarter (n = 102)	Third quarter (n = 99)	Fourth quarter (n = 94)
Oral medication	31 (28.7 %)	23 (32.4 %)	20 (20.2 %)	18 (19.1 %)
Physical therapy	60 (55.6 %)	54 (52.9 %)	54 (54.5 %)	51 (54.3 %)
Occupational therapy	43 (39.8 %)	42 (41.2 %)	41 (41.4 %)	44 (46.8 %)
Speech therapy	10 (9.3 %)	8 (7.8 %)	8 (8.1 %)	9 (8.6 %)
Other therapies	3 (2.8 %)	6 (6.0 %)	4 (4.0 %)	4 (4.3 %)
Therapeutic aids	12 (11.0 %)	5 (5.7 %)	-	1 (1.0 %)
Conventional therapy group: Antispastic medications, non-pharmacological therapies and aids				
	First quarter (n = 110)	Second quarter (n = 98)	Third quarter (n = 91)	Fourth quarter (n = 84)
Oral medication	67 (60.9 %)	66 (67.3 %)	63 (69.2 %)	58 (69.0 %)
Physical therapy	68 (61.8 %)	59 (60.2 %)	54 (54.5 %)	52 (61.9 %)
Occupational therapy	15 (13.6 %)	11 (11.2 %)	11 (12.1 %)	8 (9.5 %)
Speech therapy	5 (4.6 %)	5 (5.1 %)	5 (5.5 %)	4 (4.8 %)
Other therapies	3 (2.7 %)	5 (5.1 %)	-	1 (1.2 %)
Therapeutic aids	10 (11.0 %)	8 (8.2 %)	12 (13.2 %)	8 (9.5 %)

**Table 4:** Responder analyses at study end after 1-year of treatment

	INCO pretreated	INCO naïve	INCO total	CON	INCO pretr. vs. CON	INCO naïve vs. CON	INCO total vs. CON
Shoulder adduction/internal rotation	56.4	73.9	62.9	15.5	<0.01	<0.01	<0.01
Shoulder abduction	65.5	100	73.0	19.7	<0.01	<0.01	<0.01
Shoulder elevation	66.7	88.9	72.7	20.6	<0.01	<0.01	<0.01
Flexed elbow	78.3	92.9	83.8	26.9	<0.01	<0.01	<0.01
Forearm pronation	81.4	73.7	79.0	22.0	<0.01	<0.01	<0.01
Flexed wrist	82.1	94.7	86.2	26.6	<0.01	<0.01	<0.01
Thumb-in-palm	77.8	81.3	78.8	20.0	<0.01	<0.01	<0.01
Clenched fist	79.1	95.2	84.4	22.2	<0.01	<0.01	<0.01
Intrinsic-plus-position of the hand	73.3	100	78.9	19.5	<0.01	<0.01	<0.01

Responder rates (%); response was definded as ≥ 1-point improvement on the Ashworth Scale for all treated muscle groups at study end; Fisher´s exact test was used for group comparisons

**Table 5:** Overview of total costs by cost centers (in €)

	INCO *n* = 93	CON *n* = 83
Ambulatory medical treatment	175	217
Drugs	3,386	193
Hospitalizations (including rehabilitation measures)	40	138
Non-pharmacological therapies	1,408	998
Medical devices / aids	79	12
Nursing home care	3,089	2,203
Total direct costs	8,188	3,806
Reduction in earning capacity	2,081	988
Total costs	10,268	4,794

**Table 6:** Overview of Cost-utility ratios and ICER

Utility parameter	INCO		CON		
*Responder rate in Ashworth Score per clinical pattern*	*Responder rate*	*Cost-utility ratio*	*Responder rate*	*Cost-utility ratio*	*ICER*
Shoulder adduction / internal rotation	62.9 %	16,325 €	15.5 %	30,929 €	11,549 €
Shoulder abduction	73.0 %	14,066 €	19.7 %	24,335 €	10,271 €
Shoulder elevation	72.7 %	14,124 €	20.6 %	23,272 €	10,507 €
Flexed elbow	83.8 %	12,253 €	26.9 %	17,821 €	9,621 €
Pronated forearm	79.0 %	12,998 €	22.0 %	21,791 €	9,604 €
Flexed wrist	86.2 %	11,912 €	26.6 %	18,022 €	9,185 €
Thumb-in-palm	78.8 %	13,031 €	20.0 %	23,970 €	9,310 €
Clenched fist	84.4 %	12,166 €	22.2 %	21,595 €	8,801 €
Intrinsic-Plus-position (hand)	78.9 %	13,014 €	19.5 %	24,585 €	9,216 €
*Improvement in SF-12 dimension*	*Improvement*	*Cost-utility ratio*	*Improvement*	*Cost-utility ratio*	*ICER*
Physical Health	7.96	1,290 €	0.83	5,776 €	768 €
Mental Health	10.75	955 €	5.71	840 €	1,086 €

Incremental Cost-Effectiveness Ratio (ICER) = (Total costs INCO – Total costs CON)/(Utility value INCO – Utility value CON)
